# Seroprevalence of *Borrelia burgdorferi* in Belgian forestry workers and associated risk factors

**DOI:** 10.1186/s13071-018-2860-2

**Published:** 2018-05-02

**Authors:** Mathilde De Keukeleire, Annie Robert, Victor Luyasu, Benoît Kabamba, Sophie O. Vanwambeke

**Affiliations:** 1 Earth and Life Institute (ELI), Georges Lemaitre Center for Earth and Climate Research, Université catholique de Louvain (UCL), Louvain-la-Neuve, Belgique; 2 Pôle Epidémiologie et Biostatistique (EPID), Institut de Recherche Expérimentale et Clinique (IREC), Faculté de Santé Publique (FSP), Université catholique de Louvain (UCL), Bruxelles, Belgique; 3 Laboratory of Medical Microbiology, Université Catholique de Louvain (UCL), Bruxelles, Belgique

**Keywords:** *Borrelia burgdorferi*, Lyme disease, Seroprevalence, Serology, Tick, Exposed groups, Forestry workers, Risk, Belgium

## Abstract

**Background:**

As forest is the preferred environment for ticks, forestry workers are exposed to tick bites and tick-borne diseases. We assessed the seroprevalence of anti-*Borrelia burgdorferi* (*Bb*) antibodies and investigated, using an integrated landscape approach, the individual and environmental factors associated with the seroprevalence of *Bb* in Belgian forestry workers, a high-risk group in Belgium.

**Methods:**

A group of 310 Belgian forest workers was examined to assess the seroprevalence of anti-*Borrelia* IgG antibodies. Using principal component analysis and binary logistic regression, the joint effects of individual characteristics and environmental characteristics were examined.

**Results:**

Sixty-seven of the 310 workers were seropositive for Lyme disease (LD), leading to a seroprevalence of 21.6%. The seroprevalence was higher among forest workers visiting forests more frequently (*P* = 0.003) or who reported over 100 tick bites (*P*-value < 0.001). The intensity of tick bites and the use of protection measures against tick bites have a positive impact on LD seroprevalence while the quantity of shadow from trees at ground level had a negative one.

**Conclusions:**

This study showed that forest workers are a population at risk for LD and, by extension, at risk for various tick-borne diseases. In addition to the role of the environment, our results also showed the importance of considering exposure when predicting the risk of infection by *Bb*.

## Background

As forests and wooded areas are the preferred environment for ticks, forestry workers and other populations in close contact with outdoor environments, such as farmers, veterinarians, hunters, orienteers, mushrooms or berry pickers, are at high risk of tick bites and exposed to tick-borne diseases (TBD) [1]. Studying these groups can inform about tick-borne diseases and their spatial distribution [2].

The main vector of TBD in Europe is *Ixodes ricinus* [3]. *Ixodes ricinus* is a vector for various pathogens, including viruses (tick-borne encephalitis virus), protozoans (*Babesia* sp.) or bacteria (*Anaplasma phagocytophilum*). The most famous bacteria transmitted by ixodid ticks is the spirochete *Borrelia burgdorferi* (*Bb*) (*s.l.*), which can cause Lyme disease (LD) [3, 4]. A Belgian study reported that 12.0% of ticks were infected by *Bb* [5]. The most frequent genomospecies were *Borrelia afzelii* (55%) and *B. garinii* (21%). Another recent study showed that 17.6% of nymphs were infected, most commonly by *B. afzelii* [6]. The main clinical manifestation of LD is erythema migrans (EM) but the spirochete can cause early disseminated LD (arthritis, neuroborreliosis) and late disseminated LD, with manifestations such as acrodermatitis chronica atrophicans or Lyme arthritis [3, 7]. LD diagnosis is mainly based on clinical history and symptoms (presence of EM, facial palsy, arthritis) and serological response to *Bb*, except in the early stages [8].

Understanding the spatial variation of TBD risk and its causes is essential for disease management and prevention [9]. As the vegetation used by ticks for sheltering and questing is most abundant in forests, as well as hosts used for blood-feeding, forestry workers are particularly at risk of tick bites and TBD.

Several methods exist to assess the risk of TBD; one is the use of serological investigations in groups at high-risk of tick bites [10]. Numerous studies have estimated the seroprevalence of *Bb* in forestry workers in several European countries, but it has never been made in Belgium [11]. Moreover, as forestry workers are highly exposed to tick bites, and work in heavily wooded yet diverse environments, there is a need to better understand *Bb* transmission in a spatially explicit framework. Individual as well as environmental factors can influence the risk of infection. Indeed, several studies highlighted the importance of accounting for human behavior and land use in addition to land cover and variables describing tick habitat in epidemiological models [12–17].

This study aimed to assess the seroprevalence of *Bb* in Belgian forestry workers, a high-risk group, and to investigate, using an integrated landscape approach, which individual and environmental factors favor *Bb* infection.

## Methods

### Epidemiological study and serological examinations

**Fig. 1:**
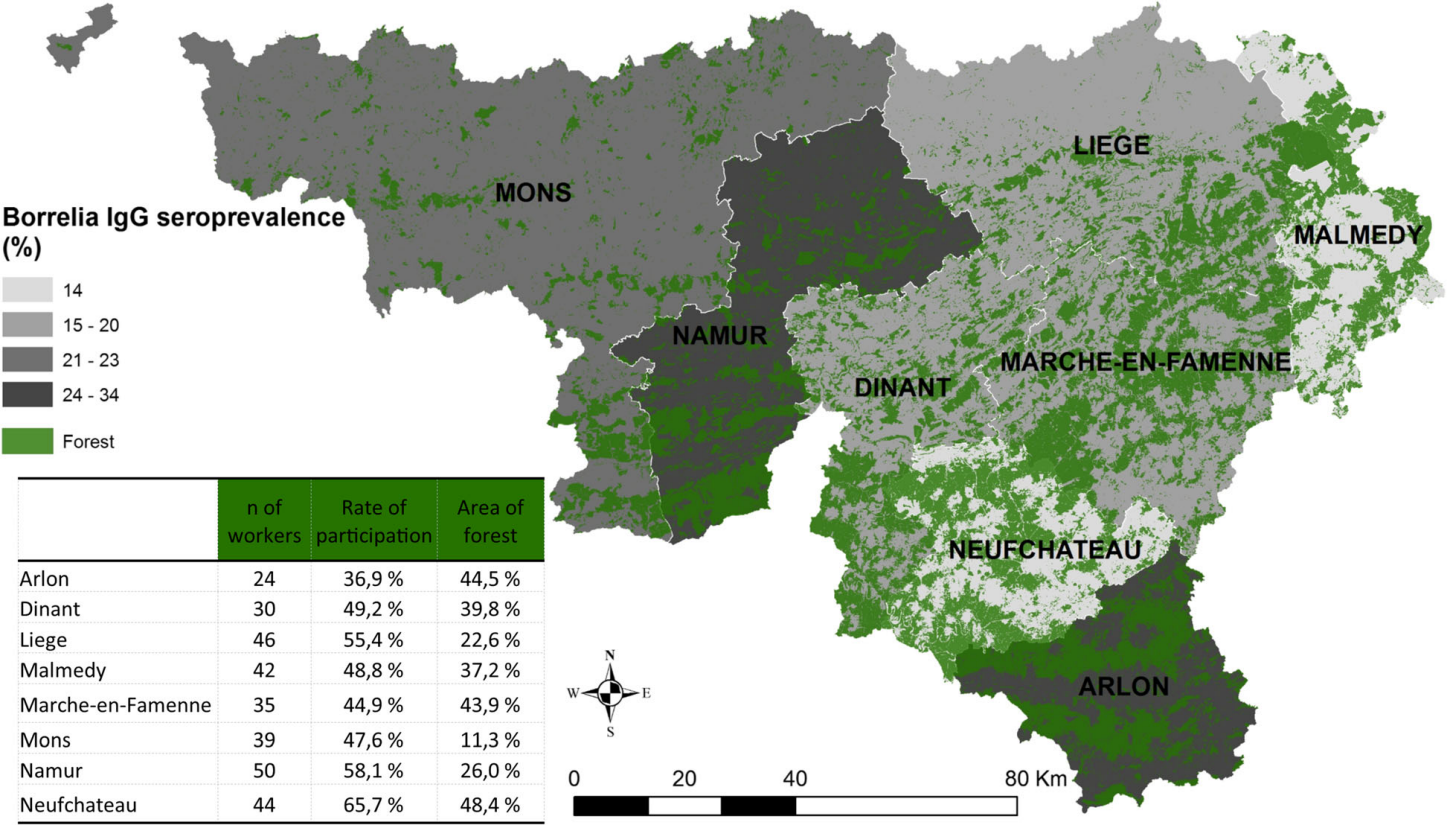
*Borrelia* IgG seroprevalence of forest workers depending on the territorial where they work

This study targeted forest workers from the Department of Nature and Forests (DNF) in the south of Belgium (Walloon region). Subjects were invited in June 2016 by their hierarchy to participate to one of the eight meetings organized in the territorial management units (TMU) (Arlon, Dinant, Liège, Malmedy, Marche, Mons, Namur and Neufchâteau) (Fig. 1). All volunteer forest workers filled in a questionnaire covering socio-demographic characteristics, exposure to tick bites and environments suitable for ticks during professional activities and leisure time, use of prevention measures, as well as details related to their potential clinical history of LD. Afterwards, a physician took blood samples to assess the presence of anti-*Borrelia* IgG antibodies. All participants signed an informed consent.

Sera were tested for the presence of anti-*Borrelia* IgG antibodies using an ELISA method at one of the two laboratories constituting the national reference center for LD, the Clinique Saint-Luc Laboratory (Brussels). The LIAISON® Borrelia IgG assay was used on the Liaison XL instrument (DiaSorin S.p.A.) to detect anti-*Borrelia* IgG. According to manufacturer recommendations, IgG ≤ 10 UI were considered negative, IgG > 15 UI positive, and the remaining values borderline. The ELISA test showed a high sensitivity (100%) with a good specificity (90%) for the detection of *Bb* infection.

### Data set

Given the structural organization of the “Service public de Wallonie”, forest workers are allocated to eight TMU within which they are working. Consequently, all independent variables were computed at the level of TMU. They are listed in Table 1.

**Table 1:** Summary statistics of environmental covariables

Variable	Descriptive statistics		
	Mean	SD	Range
Landscape composition variables			
Proportion of artificial land (%)	12.6	4.9	7.6–20.5
Proportion of wetland (%)	1.4	1.1	0.7–4.2
Proportion of grassland (%)	30.0	5.7	21.2–39.5
Proportion of arable land (%)	18.5	12.8	4.8–43.1
Proportion of permanent crops (%)	0.4	0.5	0–1.6
Proportion of fallow land (%)	0.4	0.2	0.2–0.7
Proportion of hardwood forest (%)	10.6	6.4	0.9–19.5
Proportion of conifer forest (%)	9.6	8.1	0.3–22.9
Proportion of forest (unspecified type) (%)	13.9	5.8	3.2–21.9
Public forest (%)	32.0	19.0	2.2–67.8
Landscape configuration variables			
Area-weighted mean shape index (-)	2.2	0.2	2.0–2.7
Area-weighted mean patch fractal dimension (-)	1.30	0.01	1.29–1.31
Edge density (m/ha)	150.8	46.2	97.2–216.0
Patch density number (#/ha)	0.2	0.1	0.1–0.4
Wildlife variables			
Boar density per TMU (*n*/km^2^)	4.4	1.4	2.4–7.3
Roe deer density per TMU (*n*/km^2^)	7.3	1.4	6.1–10.1
Red deer density per TMU (*n*/km^2^)	2.1	1.2	0–3.5
Boar shot per TMU (*n*/km^2^)	3.7	1.8	1.2–6.7
Roe deer shot per TMU (*n*/km^2^)	3.2	0.6	2.7–4.3
Red deer shot per TMU (*n*/km^2^)	0.9	0.5	0–1.4
Belgian forest inventory variables			
Natura 2000	0.3	0.06	0.2–0.5
Slope	6.3	1.5	3.9–8.9
ST_3_ - < 5%	17.9	2.3	13.9–22.1
ST_3_ - 5–24%	24.0	4.3	15.6–29.1
ST_3_ - 25–49%	15.2	3.0	10.6–19.7
ST_3_ - 50–75%	7.1	1.2	5.6–8.9
ST_3_ - > 75%	3.1	1.0	1.4–5.5
ST_3–9_ - < 5%	9.1	1.8	6.9–12.4
ST_3–9_ - 5–24%	16.2	3.7	10.2–24.2
ST_3–9_ - 25–49%	16.3	4.6	9.3–21.4
ST_3–9_ - 50–75%	11.4	4.3	5.0–17.2
ST_3–9_ - > 75%	8.5	3.0	4.6–12.5

*Abbreviations*: *SD* Standard deviation, *ST*_*3*_ The amount of shadow at ground level produced by trees smaller than 3 m, *ST*_*3–9*_ The amount of shadow at ground level produced by trees of 3–9 m high, *TMU* Territorial management units

Landscape composition and configuration in each TMU were measured using the Wallonia land cover map [Carte d’occupation du sol de Wallonie (2007) from the “Service public de Wallonie” (Copyright-SPW-n°140225-1407)]. As detailed in a previous study [15], the proportions of several land cover types were calculated for each TMU (Table 1). Landscape configuration was described by using various indices focusing on an aggregated forest class (forest and semi-natural habitats) (Table 1).

The numbers of red deer, roe deer and wild boar and the number of red deer shot, roe deer shot and wild boar shot by directions from 2002 to 2011 were retrieved from the "Department of Hunting and Fishing of Wallonia". We calculated the mean number of animals and the mean number of animals shot by TMU over the 2002–2011 period because the date of infection is unknown. To compare with previous studies, we divided those numbers by the forested area in the direction and obtained the density of game and game shot by TMU [13, 15].

Data from the Belgian forest inventory, carried out since 1994, were also used. The sampling design and variables collected have been described previously [18]. Briefly, sampling points are regularly spaced at distances of 1 km longitudinally and 500 m latitudinally. In each sampling unit, information was collected about characteristics of trees, flora, management, game presence, etc. Four variables were selected here: the mean slope, the presence of Natura 2000 zones and the amount of shadow at ground level produced by trees smaller than 3 m (ST_3_) and 3–9 m high (ST_3–9_). These two last variables were divided into five classes: < 5%, 5–24%, 25–49% 50–75% and > 75%. At TMU level, we computed the mean slope and aggregated all other inventory data as per the equation:

$$ \boldsymbol{X}=\frac{\boldsymbol{number}\ \boldsymbol{of}\ \boldsymbol{points}\ \boldsymbol{in}\ \boldsymbol{the}\ \boldsymbol{class}\ \boldsymbol{for}\ \boldsymbol{the}\ \boldsymbol{management}\ \boldsymbol{unit}}{\boldsymbol{total}\ \boldsymbol{number}\ \boldsymbol{of}\ \boldsymbol{points}\ \boldsymbol{in}\ \boldsymbol{the}\ \boldsymbol{mangement}\ \boldsymbol{unit}} $$

### Statistical methods

The questionnaire provides rich individual information related to the exposure to tick bites, to favorable environments during professional activities and leisure time, and the use of prevention measures were available. A principal components analysis (PCA) was conducted on individual characteristics to summarize the data. Propensity scores were calculated for each dimension. Missing values were handled beforehand by imputation performed with multiple linear methods. All data were standardized to avoid scale dependence. The absolute contribution of original variables to the dimensions was used to interpret the PCA axes. A contribution was considered as high when it was higher than 9.1 (100/number of variables). Propensity scores were computed for each dimension and rescaled to a score varying between 0 and 10.

Forest workers are grouped in TMU and so share environmental characteristics. The necessity of a multilevel model was checked by using the intraclass correlation coefficient (ICC). It was unnecessary to account for the nested structure because the within-group variance was higher than the between-groups variance (ICC = 0).

Our dependent variable was binary (seropositive, yes or no) and we therefore used binary logistic regression. Variables were standardized to avoid scale dependence. Variables with a univariate *P*-value > 0.20 were removed to reduce multiple testing. If a strong correlation was noticed between explanatory variables (r > 0.90 or Variance Inflation Factor > 10), one of the two was eliminated to avoid multicollinearity. Then, variables with a *P*-value > 0.10 were considered as candidates for elimination. Afterwards, chi-square likelihood ratio tests with a *P*-value > 0.10 and the Schwartz criterion were used to remove variables, if there was no loss of likelihood.

Study results are presented as odds ratio (OR) with 95% confidence intervals (CI). The statistical significance level was set to 0.05. Epi-Info version 7 and SPSS 20.0 were used to calculate Chi-square Cochran-Armitage trend tests and Fisher exact tests.

## Results

### Study group

Among the 608 workers of the DNF, 315 accepted to participate to the survey. The study group consisted of 297 male and 18 female forest workers, representing a global participation rate of 52% (Table 2). Five individuals, working for the fishing department, were excluded from the analysis because they cannot be attributed to a specific TMU. Therefore, this study included 310 forest workers (293 men and 17 women). The rate of participation was higher in Namur (50 of 86), Neufchâteau (44 of 67) and Liège (46 of 83), independently of the percentage of the unit occupied by forest (Fig. 1). Forests covered 48.4% of the TMU of Neufchâteau, but only 11.3% of the TMU of Mons. The median age of workers was 49 years, with a minimum of 24 years and a maximum of 65 years. 48.1% of participants were older than 50 years, and 50.7% worked in silviculture for more than 20 years. The duration of employment in silviculture and age were highly correlated (*r* = 0.77, *P* < 0.01).

**Table 2:** Demographic and exposure characteristics of the 310 forest workers (Wallonia, Belgium)

Characteristic		Subjects	
		*n*	%
Gender	Male	293	94.5
	Female	17	5.5
Age	Less than 50 years	161	51.9
	50 years or more	149	48.1
Duration of employment in silviculture	Up to 20 years	153	49.3
	More than 20 years	157	50.7
Frequency of forest visits	Less than 3 times per week	36	11.6
	3 times per week or more	274	88.4
Mean duration of forest visits	Up to 5 hours	104	33.5
	1 day	206	66.5
Use of protective measures	+	182	58.7
	-	128	41.3
Use of protective clothes	+	160	51.6
	-	150	48.4
Use of repellents	+	68	21.9
	-	242	78.1
Tick bites during work	+	294	94.8
	-	16	5.2
Number of tick bites	0–10	88	29.8
	11–100	141	47.8
	More than 100	66	22.4
Frequency of tick bites	Less than 1 per month	141	48.3
	1 per month or more	151	51.7
Method to remove the tick	Use of tick tweezer	210	67.7
	Use of tweezer	46	14.8
	Use of fingers	39	12.6
	Others	15	4.8
History of Lyme disease signs or symptoms	Erythema migrans	23	26.4
	Articular symptoms	29	33.3
	Articular and neurological symptoms	21	24.1
	Others	14	16.1
Cause of Lyme borreliosis	Tick Bite	57	27.3
	Bacteria	115	55.1
	Other causes	36	17.6

A total of 88.4% of participants frequented forests at least three times a week as part of their professional activities. The duration of a visit was a whole day for 66.5% of workers. Protective measures, such as protective clothing, gaiters and repellents, are used by 58.7% of participants when visiting forests.

A history of tick bite while working was reported by 94.8% of forest workers (*n* = 294). More than 65% reported that they got bitten over 11 times while working, and 66 workers (21.3%) got bitten over 100 times. 51.7% of workers reported getting bitten at least once per month. The number and frequency of tick bites were significantly positively correlated (*r* =0.67, *P* < 0.01). The number of bites increased with duration of employment (*r* = 0.32, *P* < 0.01). Most workers (67.7%) reported the use of tick forceps to remove ticks attached to their body. However, they also used tweezers, fingers or other methods such as soap. A history of LD signs was reported by 87 workers (28.1% of the study group): 23 had suffered from an EM and 29 from articular symptoms. A large proportion of the study subjects (66.1%) knew the cause of LD: 55.1% specified that it is caused by a bacteria and 27.3% by a tick bite.

### Seroprevalence

Anti-*Borrelia* IgG antibodies were detected in 67 forest workers (21.6%). The test result was borderline in 1.3% (*n* = 4) of the study group. These borderline results were considered as negative in the analyses.

The seroprevalence was highest in Namur and in Arlon with 34.0 and 29.0%, respectively (Fig. 1). Malmedy and Neufchâteau had the lowest seroprevalence, 14% (Fig. 1). The proportion of positive test results increased with age (from 0 to 40.0%) and with the duration of employment (from 6.8 to 36.5%) (Table 3). A higher seroprevalence was observed in workers older than 50 years (OR = 3.89; 95% CI: 2.14–7.08; *P* < 0.001). People working in silviculture for over 20 years had a seropositivity rate higher than those with less than 20 years (OR = 3.74). The seroprevalence was higher among forest workers visiting forest more frequently (three times per week or more) (OR = 11.11; 95% CI: 1.49–82.64; *P* = 0.003). Workers visiting forests for entire days also had a higher seroprevalence, but this difference was not statistically significant (*P* = 0.11).

**Table 3:** Univariate association between characteristics of foresters and the presence anti-*Borrelia* IgG antibodies in 310 forest workers (Wallonia, Belgium)

Factor		OR	95% CI	*P*-value
Age	50 years or more	3.89	2.14–7.08	< 0.001
	Less than 50 years	1	–	–
Duration of employment in silvicultural field	More than 20 years	3.74	2.04–6.85	< 0.001
	Up to 20 years	1	–	–
Frequency of forest visits	3 times per week or more	11.11	1.49–82.64	0.003
	Less than 3 times per week	1	–	–
Mean duration of forest visits	1 day	1.64	0.89–3.02	0.11
	Less than 1 day	1	–	–
Use of protective measures	+	1.14	0.65–1.98	0.64
	-	1	–	–
Use of protective clothes	+	1.20	0.70–2.07	0.50
	-	1	–	–
Use of repellents	+	1.73	0.94–3.18	0.08
	-	1	–	–
Tick bites during work	+	4.34	0.56–33.48	0.12
	-	1	–	–
Number of tick bites	More than 100	5.94	3.24–10.90	< 0.001
	Less than 100	1	–	–
Frequency of tick bites	1 per month or more	2.90	1.60–5.25	< 0.001
	Less than 1 per month	1	–	–
Use of tick tweezer to remove tick	+	0.63	0.36–1.12	0.11
	-	1	–	–
Use of tweezer to remove tick	+	0.86	0.39–1.89	0.71
	-	1	–	–
Use of fingers to remove tick	+	2.62	1.29–5.37	0.006
	-	1	–	–
Knowing the cause of Lyme borreliosis	+	0.91	0.50–1.65	0.75
	-	1	–	–

*Abbreviation*: *CI* Confidence interval

People using protective measures, protective clothing or repellents had a higher seroprevalence, but this difference was not significant (Table 3). Seroprevalence was higher in individuals who reported over 100 tick bites (OR = 5.94; 95% CI: 3.24–10.90; *P* < 0.001). A higher seroprevalence was observed in workers getting bitten at least once per month while working (OR = 2.90; 95% CI: 1.60–5.25; *P* ≤ 0.001) and in individuals who removed ticks with fingers (OR = 2.62; 95% CI: 1.29–5.37; *P* = 0.006). Individuals who used tick tweezers or tweezers to remove ticks had lower odds to be seropositive (Table 3). Only 37 forest workers (12% of the study group) of the 87 workers reporting a history of LD were seropostive. Knowing the cause of LD was not associated with seroprevalence (OR = 0.91; 95% CI: 0.50–1.65; *P* = 0.75).

### Individual epidemiological characteristics

To characterize the exposure of forests workers, a PCA was conducted on eleven individual characteristics: gender, age, duration of employment in silviculture, frequency of forest visits, duration of forest visits, use of protective measures, use of protective clothing, use of repellents, tick bites during work, number of tick bites and frequency of tick bites.

**Fig. 2:**
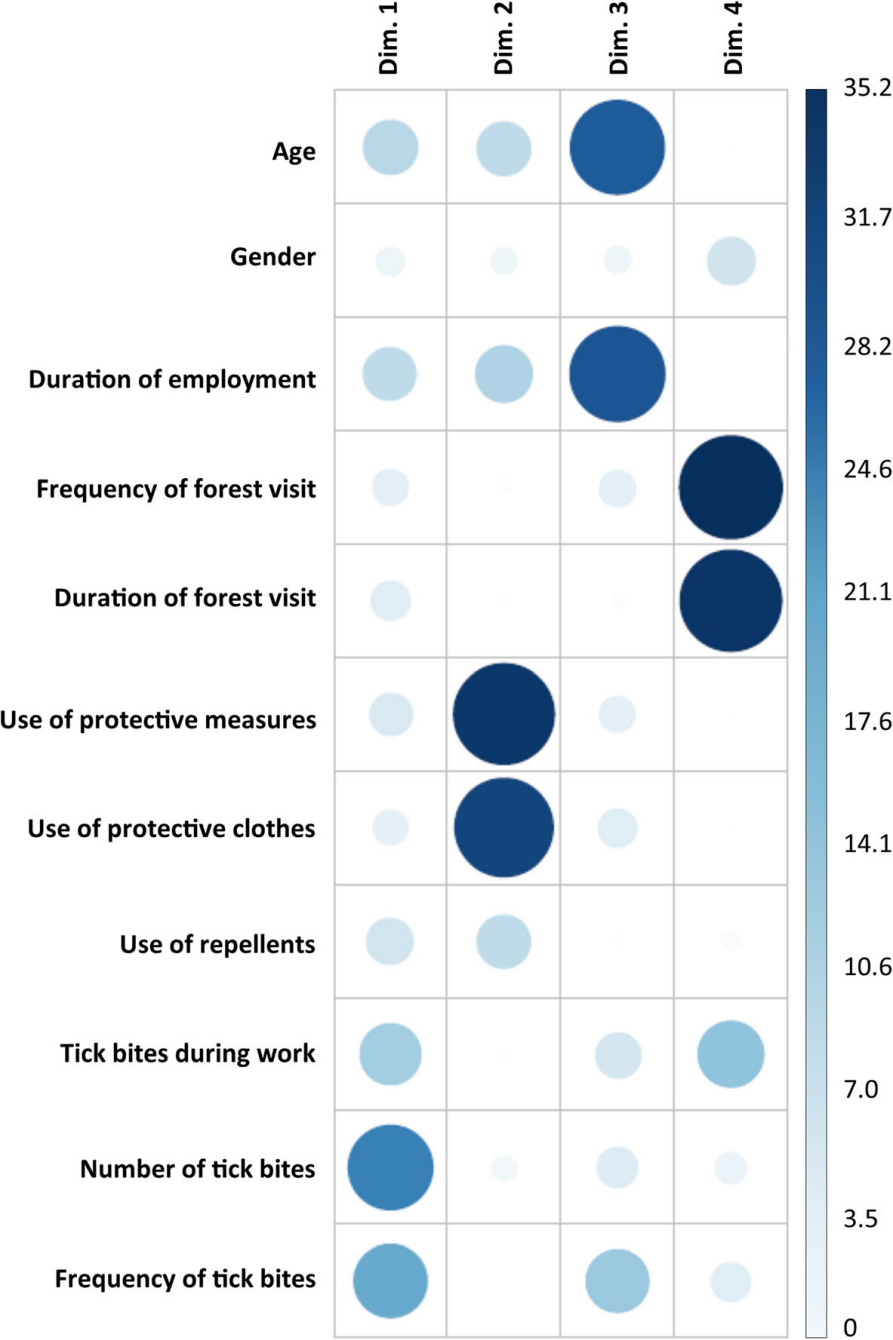
Absolute contributions of variables to dimensions (The darker the blue and bigger the circle, the higher the weight of the variable in a dimension)

The PCA showed four significant axes explaining 68.5% of the total inertia (24.7%, 19.4%, 12.8% and 11.5% inertia for dimensions 1, 2, 3 and 4, respectively). Tick bites during work, number of tick bites and frequency of tick bites were the variables contributing the most to the first dimension (Fig. 2). The first dimension was positively correlated with tick bites during work (0.58), the number of tick bites (0.80) and frequency of tick bites (0.70). Therefore, we could interpret the first dimension as “Intensity of tick bites”.

The use of protective measures, of protective clothing and of repellents were negatively correlated with the second dimension (-0.85, -0.83 and -0.44, respectively) (Fig. 2). The second dimension could be interpreted as “Use of protection against tick bites”. As the third dimension was characterized and correlated with age (0.64) and duration of employment in silviculture (0.65), it could be interpreted as “Duration of exposure” (Fig. 2).

The most important variables for the fourth dimension related to the intensity of forest visits: frequency and duration of forest visits were positively correlated with the dimension (0.67 and 0.66, respectively). The fourth dimension could be interpreted as “Intensity of forest visits”.

Gender was represented by the fifth dimension. However, as our study group included very few women, we can leave this variable aside.

**Fig. 3:**
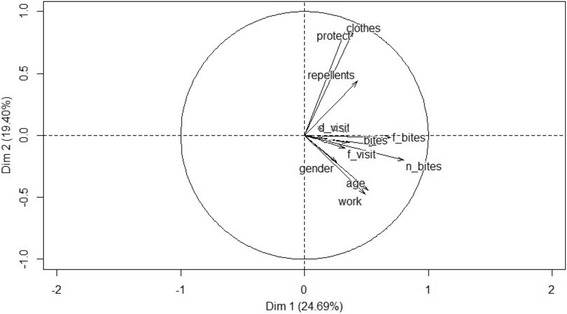
Biplot of variables: Age, Gender, Work in silvicultural field (work), Frequency of forest visit (f_visit), Duration of forest visit (d_visit), Use of protective measures (protect), Use of protective clothing (clothes), Use of repellents (repellents), Tick bites during work (bites), Number of tick bites (n_bites), Frequency of tick bites (f_bites)

The use of protection was correlated with the duration of exposure (age and work in silviculture) and gender (Fig. 3). Bivariate logistic regressions showed a strongly significant relation between the presence or absence of anti-*Borrelia* IgG antibodies and the two first dimensions: workers with a higher intensity of tick bites or using less protection against tick bites were significantly more often seropositive (Table 4).

**Table 4:** Bivariate logistic regressions between the presence of anti-*Borrelia* IgG antibodies and the four dimensions among forest workers (Wallonia, Belgium)

Presence or absence of anti-*Borrelia* IgG antibodies	OR (95% CI)	*P*-value
Dim 1: Intensity of tick bites	3.34 (2.24–4.96)	< 0.0001
Dim 2: Use of protection against tick bites	1.37 (1.04–1.80)	0.02
Dim 3: Duration of exposure	1.14 (0.87–1.49)	0.34
Dim 4: Intensity of forest visits	1.13 (0.85–1.49)	0.40

*Abbreviation*: *CI* Confidence interval

### Individual environmental characteristics

Table 5 presents the association between environmental characteristics and the presence of anti-*Borrelia* IgG antibodies. The proportion of conifer forest and the proportion of inventory points where the shadow of trees 3–9 m high was less than 5% were significantly negatively correlated with the presence of *Borrelia* infection. A significant positive correlation was noted for all other classes of the shadow of trees 3–9 m high and for the proportion of point where the shadow of trees smaller than 3 m high fluctuated between 25–75%.

**Table 5:** Bivariate logistic regressions between the seropositivity for *Borrelia* infection and environmental variables

Standardized variables	OR (95% CI)	*P*-value
Proportion of arable lands (%)	1.26 (0.97–1.65)	0.09
Proportion of grassland (%)	0.76 (0.58–1.01)	0.05
Proportion of forest (unspecified type) (%)	0.86 (0.63–1.08)	0.16
Proportion of hardwood forest (%)	1.29 (0.98–1.70)	0.07
Proportion of conifer forest (%)	0.74 (0.55–0.98)	0.04
Public forest (%)	0.82 (0.62–1.08)	0.16
Roe deer density shot per direction (*n*/km^2^)	0.77 (0.59–1.00)	0.05
ST_3_ - 5–24%	1.36 (1.01–1.83)	0.04
ST_3_ - 25–49%	1.45 (1.10–1.92)	0.09
ST_3–9_ - < 5%	0.70 (0.52–0.94)	0.02
ST_3–9_ - 25–49%	1.39 (1.05–1.86)	0.02
ST_3–9_ - 50–75%	1.40 (1.06–1.84)	0.02
ST_3–9_ - > 75%	1.34 (1.02–1.77)	0.04

*Abbreviations*: *ST*_*3*_ The amount of shadow at ground level produced by trees smaller than 3 m, *ST*_*3–9*_ The amount of shadow at ground level produced by trees of 3–9 m high

### Factors associated with the seroprevalence of *Bb*

Table 6 presents the results of univariate and multivariate regressions. Dimensions 1 and 2 were associated with an increase in risk. Dimension 1, representing the intensity of tick bites, had a greater impact on the risk of being seropositive. The seroprevalence of *Bb* was also associated with the shadow of trees 3–9 m high - Less than 5%. This variable measures the amount of tree shadow on the ground: an increase in the variable relates to a more open landscape where larger fractions of the ground surface receive sunlight. An increase in ST_3–9_ - Less than 5% was associated with a decrease in *Bb* seroprevalence.

**Table 6:** Results from the multivariate logistic regression

	Univariate	Multivariate	
	adjOR	OR (95% CI)	*P*-value
Z_Dim 1_ - _Intensity of tick bites_	3.59 (2.36–5.47)	3.66 (2.40–5.57)	< 0.0001
Z_Dim 2 - Use of protective measures_	1.56 (1.15–2.13)	1.57 (1.15–2.15)	0.004
Z_ST3–9 - < 5%_	0.70 (0.52–0.94)	0.71 (0.51–0.98)	0.039

*Abbreviations*: *adjOR* Adjusted OR, *CI* Confidence interval

## Discussion

### Study group

In this study, we enrolled forest workers, a group of land users at high risk of tick bites. It is the first time that sera from forest workers were analyzed in Belgium. It allowed us to confirm that they are highly exposed to LD. It is important to note that our sample is not representative of the normal Belgian population and therefore our results cannot be extended to the general population. Indeed, we specifically targeted a population highly exposed to tick bites. Moreover, forest workers usually have a higher level of knowledge about ticks and TBD and are more likely to protect themselves: about 60% of forest workers used protective measures.

Our sample may have some potential biases. Forest workers with greater awareness of LD may be more likely to participate to a study like the present one. Others bias can also exist: some forest workers may not be able to participate due to lack of time or because they did not receive the information about the survey. Distance cannot be considered as a bias as a meeting was organized in each TMU. The survey was organized during working hours and participants were reimbursed for travel fees. We can thus consider our sample representative and our general conclusions valid.

Missing data were not a problem. There were only three missing values, for the variable “frequency of tick bites”, which were imputed using multiple linear methods. Sensitivity analyses comparing results with and without imputation indicated no difference in results.

### Seroprevalence

The seroprevalence of *Borrelia* IgG was 21.6%. A study conducted in the same geographical region and using the same serological test showed a seroprevalence of 2.6 and 2.9% for a population of urban and rural blood donors, respectively [19]. In the same area, the seroprevalence of *Bb* of veterinarians and famers, other workers exposed to TBD, was estimated at 5.4% [15, 19]. The comparison with these control groups allows us to state that forest workers are at higher risk than those three populations (Chi-square Cochran-Armitage trend test *P*-value < 0.01). Similarly to other studies, our sample highlighted an over-representation of positive serologies for *Bb* in forestry workers [11, 20].

Many other studies have been conducted on the *Bb* seroprevalence among forest workers. However, a range of techniques were used (ELISA with or without western blot confirmation, detection of IgG and IgM antibodies), which limits comparisons. Studies based on the detection of IgG with ELISA reported seroprevalence rates among forest workers of 30.6% in Germany, 35% in Switzerland, 24.3% in Italy, 29.2% in Slovakia and 23.8% in Slovenia [2, 21–24]. Other studies based on IgG and IgM reported seroprevalences of 14.1% in France and between 18% and 52% in southwest Germany [25, 26].

A study of patients diagnosed at various stages of LD reported that most patients were infected in the central and south-eastern part of Belgium [4], which is consistent with our results. Another study based on data from the Scientific Institute of Public Health and georeferenced infections according to the municipality of infection indicated high concentrations of infections in Dinant, Namur and Arlon [14].

Our distribution is also in line with the distribution of positive results of laboratory tests reported by a sentinel network of laboratories over the period 2000–2015 [27]: a low incidence of *Bb* positive results in the western and eastern parts of Wallonia and a high incidence in the south parts of the provinces of Namur and Luxemburg. However, those data differ from ours as they are spatially linked to the place of residence, which does not always reflect the place of infection.

Little information is available regarding the prevalence of *Borrelia* in ticks in Belgium. A Belgian study reported that 12.0% of tick were infected by *Bb* [5]. At least five species are found in Belgium: *B. afzelii*, *B. garinii*, *B. burgdorferi* (*s.s.*), *B. spielmanii* and *B. valaisiana* [5, 28]. A study showed in 2014 that the most prevalent *Borrelia* species was *B. garinii* (54% of infected ticks), followed by *B. valaisiana* (27%) and *B. burgdorferi* (*s.s.*) and *B. afzelii* (9%) [29]. Because some *Borrelia* species are structured ecologically into clusters that are host-specific [30], these host associations are likely to have an impact on the geographical population structure of the genomospecies. Another recent study showed that 17.6% of analyzed nymphs were infected and the most common is *B. afzelli* [6].

As observed in other studies, seroprevalence was higher as age and duration of employment in silviculture increased [2, 19–21, 23, 31–33]. This is consistent with a prolonged exposure within environments favorable for ticks. Moreover, seropositivity was significantly higher for workers frequently visiting forest. This is also closely associated with the exposure to tick bites in areas favorable for ticks. This was already showed in Slovakia [2].

Half of the forest workers reported wearing protective clothing, and comparable rates were described in similar studies [34, 35]. 21.9% of forest workers reported the use of repellents, which is similar to another study [36].

A total of 94.8% of forest workers reported getting tick bites while working. Similar proportions were reported in other studies concerning forestry workers: 83% in France, 87% in Italy and 95% in Poland [24, 25, 37]. Seroprevalence was higher for workers reporting over 100 tick bites than for those reporting fewer.

The group of workers using tick tweezers or tweezers to remove ticks had a lower seroprevalence than others but this difference was not significant. However, using fingers to remove ticks was a significant risk factor. This technique may not ensure removal of the entire tick.

### Factors associated with the seroprevalence of *Bb*

Three major elements associated with the seroprevalence of *Bb* in forest workers were highlighted. First, the first dimension, representing the intensity of tick bites, was significantly positively associated with the seroprevalence of *Bb*, as shown by univariate regression. Indeed, forest workers exposed to a higher intensity of tick bites (more tick bites while working, higher number and frequency of tick bites) are more at risk of being bitten by ticks and therefore at risk of infection by *Bb*. The association between seroprevalence and history of tick bites was demonstrated by another study [33].

Secondly, the seroprevalence of *Bb* was significantly associated to the second dimension, interpreted as the use of protective measures. The variables describing the use of protective measures, of protective clothing or of repellents, were negatively correlated with the dimension. Forest workers using less protection against tick bites were more likely to be seropositive to *Bb* as they were more likely to be bitten by ticks. This relation was shown in another study [2]. However, our bivariate analysis showed that people using protective measures, protective clothing or repellents had a higher seroprevalence, though the relation was not significant. Because our data showed that people using protective measures also reported more tick bites, we can hypothesize that these people used more protection as a consequence of frequent bites.

The two first dimensions of our PCA were significant in the multivariate model of the presence or absence of anti-*Borrelia* IgG antibodies. They showed the importance of considering exposure when predicting the risk of infection by *Bb*. Indeed, as shown elsewhere, exposure is an essential component of risk and is important in predicting the risk of tick-borne diseases [12–14, 16].

Thirdly, our results indicated that the presence of anti-*Borrelia* IgG antibodies is not only related to the exposure of forest workers but also to the landscape. Indeed, our model showed a significant negative impact of ST_3–9_ - Less than 5% on the seroprevalence of *Bb*. An increase of ST_3–9_ - Less than 5% relates to a more open landscape where there is little shadow and a low density of trees. Forests form a microclimate where variations of temperature and wind speed are decreasing and where the moisture is higher. Therefore, an open landscape is less favorable for ticks because of their low desiccation resistance [38, 39].

Because we worked at the management unit level, it is difficult to establish a clear link between the environment and the seroprevalence of forest workers. The effect of landscape on the risk can be attenuated if forest workers were associated to landscapes poorly represented by the general, broad scale of the TMU. However, working at a finer scale is difficult because forest workers are very mobile. More precise data could be collected in future studies by using a geolocation system.

In Belgium, the extent of forest is stable but its characteristics change over time. In this study however, we cannot account for the short- and long-term dynamics of the forest, since the timing of infection by *Bb* is unknown. We considered the characteristics of forests at a precise date, that of the land use map and the forest inventory. We used the forest inventory to describe the forest as precisely as possible with existing data. However, even with such precise information, it cannot be excluded that a variable is a proxy of several aspects preferred by ticks and/or their hosts: undergrowth, litter, soil and litter humidity, for example. This is nearly always encountered in studies of environmental predictors of zoonotic diseases, in which no environmental descriptor can be easily associated to a specific aspect of tick or host habitat [40]. An extreme example of an environmental descriptor proxying a broad range of environmental characteristics is the remotely sensed Normalised Vegetation Difference Index (NDVI), widely used in tick-borne disease studies.

In our study, the seroprevalence was not directly associated to forested landscape. The amount of forest in the TMU was not associated to the risk of seropositivity for forest workers, who are intensively exposed to the forested environment regardless. Indeed, the management unit of Arlon and Neufchâteau have quite the same amount of forest but the seroprevalence was higher in Arlon (Fig. 1). This difference may be explained by the fact that forest workers from Arlon use more protective measures than those from Neufchâteau. Therefore, when studying seroprevalence, tick presence and abundance as well as human behavior have to be considered.

Wildlife and wildlife management were not associated to the presence of anti-*Borrelia* IgG antibodies in our study. Although deer are the most important hosts maintaining *Ixodes ricinus* tick populations in Europe [41], our data only targeted a small panel of hosts: red deer, roe deer and wild boar, at a coarse spatial and temporal resolution. This could be reassessed with more detailed and precise data.

Associating landscape factors to seroprevalence is further challenged by the unknown length of the persistence of anti-*Borrelia* antibodies [4, 42]. The temporality of tick bites is not known, and thus cannot be situated in the landscape with certainty. Uncertainty arises in our study in relation to landscape change or occupation changes for the participants. However, because of a strict framework defining land use, the landscape is quite stable in Belgium. Forest workers do not change their employment regularly: 90% of the volunteers worked in the silviculture field more than five years. Another source of uncertainty relates to participants getting bitten during leisure time. It was assumed that exposure mostly related to professional activities for the participants, and that exposure associated to leisure time was considered as negligible. The territorial units used in the study are broad and may have prevented identifying landscape-level association with infectious environments.

## Conclusions

Tick-borne diseases are present in forests of Belgium, as shown by this sero-epidemiological study performed on forestry workers. For the first time, *Bb* seroprevalence in Belgian forest workers was assessed. The objective was to assess the seroprevalence of *Bb* and to determine individual and environmental factors associated with the seroprevalence of *Bb* in forest workers. We showed the importance of considering exposure and the environment when predicting the risk of infection by *Bb*. Our results showed that forest workers are a population at risk for LD and, by extension, at risk for various TBD. In order to protect forest workers against LD and TBD, it is advisable to raise their awareness about the disease and the transmission path but particularly about the means of prevention: wearing protective clothing, applying repellent, tick check, and bathing or showering within two hours of being outdoors. Indeed, for now, prevention is the only measure to reduce TBD risk. Reducing the risk of tick bites can prevent LD and other tick-borne diseases.

## Abbreviations

*Bb*: *Borrelia burgdorferi*
ICC: Intraclass correlation coefficient
LD: Lyme disease
OR: Odds ratio
PCA: Principal components analysis
ST_3_: Amount of shadow at ground level produced by trees smaller than 3 m
ST_3–9_: Amount of shadow at ground level produced by trees 3–9 m high
TBD: Tick-borne diseases
TMU: Territorial management units
